# Assessment of PGC1α-FNDC5 Axis in Granulosa Cells of PCOS Mouse Model

**Published:** 2018

**Authors:** Shabnam Bakhshalizadeh, Farzaneh Rabiee, Reza Shirazi, Kamran Ghaedi, Fardin Amidi, Mohammad Hossein Nasr-Esfahani

**Affiliations:** 1- Department of Anatomy, School of Medicine, Zanjan University of Medical Sciences, Zanjan, Iran; 2- Department of Cellular Biotechnology, Cell Science Research Center, Royan Institute for Biotechnology, ACECR, Isfahan, Iran; 3- Cellular and Molecular Research Center, Iran University of Medical Sciences, Tehran, Iran; 4- Department of Anatomical Sciences, Faculty of Medicine, Iran University of Medical Sciences, Tehran, Iran; 5- Cell and Molecular Biology Division, Biology Department, School of Sciences, University of Isfahan, Isfahan, Iran; 6- Department of Infertility, Shariati Hospital, Tehran University of Medical Sciences, Tehran, Iran

**Keywords:** FNDC5, Granulosa cells, Infertility, Metabolic disorder, PGC1α, Polycystic ovarian syndrome

## Abstract

**Background:**

Polycystic ovarian syndrome (PCOS) is a metabolic and endocrine disorder which is characterized by hyperandrogenism, anovulation or oligomenorrhea and polycystic ovarian morphology. It is believed that modulation in metabolism of granulosa cells of PCOS patients may lead to infertility. One of the metabolic modulators is FNDC5 and its cleaved form, irisin. The axis of PGC1α-FNDC5 pathway is one of the main factors affecting cellular energy balance the purpose of this study was to evaluate this pathway in granulosa cells derived from PCOS mice model in comparison with control group.

**Methods:**

In the present study, PCOS mouse model was developed by injection of dehydroepiandrosterone (DHEA) hormone in 20 mice for a period of 20 days. Also, 20 uninjected mice were used as the control. Meanwhile, a vehicle group consisted of mice which received daily subcutaneous sesame oil injection (n=20). Relative expressions of PGC1α and FNDC5 in granulosa cells were evaluated by RT-qPCR. Analysis of gene expressions was calculated by the ΔΔCT method and the relative levels of mRNA were normalized to GAPDH transcript levels. Differences in genes expression among three groups were assessed using one-way ANOVA, Tukey’s Post Hoc test.

**Results:**

Our results showed that expression of FNDC5 was significantly reduced in granulosa cells of DHEA-induced PCOS mice compared with control and vehicle groups (p<0.05), while there was no significant differences in PGC1α expression among different groups.

**Conclusion:**

Down regulation of FNDC5 transcript level may contribute in metabolic disturbance of granulosa cells derived from PCOS ovary apart from PGC1α levels which remained unchanged.

## Introduction

Polycystic ovarian syndrome (PCOS) is considered as one of the most common metabolic and endocrine disorders that affect premenopausal women at reproductive age with a prevalence of %5 to 10 (1). PCOS is defined with two of the three following criteria: chronic anovulation or oligomenorrhea, clinical or biochemical hyper-androgenism, and polycystic ovarian morphology (2, 3). Insulin resistance is considered as the main metabolic aspect of PCOS and is aggravating by obesity (4, 5). Regarding the metabolic aspect of PCOS, this syndrome appears to be more complex than purely a reproductive disorder. As stated above obesity is considered as the driver of metabolic syndrome and accounts for the most important medical problems of the 21st century (6). One of the key genes in regulating the cellular metabolism and balance of the energy is peroxisome proliferator-activated receptor gamma coactivator 1-alpha (PGC1α) (7). This factor is responsible for mitochondrial and peroxisomal biogenesis which ultimately regulates the oxidation of macromolecules. PGC1α acts as a transcription factor and is involved in regulation of the expression of several target genes as fibronectin type III domain-containing protein 5 (FNDC5). Irisin, a secretory type of FNDC5, is released from the muscle into circulation, induces the browning of white adipose tissue and thereby increases thermogenesis and reduces insulin resistance (8). Irisin has been evaluated to act against obesity, insulin resistance, type 2 diabetes mellitus and the metabolic syndrome (9). Furthermore, serum irisin levels have been shown to have a positive correlation with parameters of lipid metabolism and glucose homeostasis (10). Despite extensive research in this filed, in the past decade, the physiological roles of FNDC5/irisin remained obscure and still some questions have not been answered (11, 12). According to the role of irisin in browning of white adipose tissue and its possible function in reducing insulin resistance, several researchers have assessed the degree of FNDC5 expression in different tissues in pathogenic states related to metabolic disorders including PCOS (13–15). Since metabolic disorders are multifactorial diseases and are affected by a number of factors such as ethnicity, social and environmental factors such as climate, diet and life style, this study aimed to evaluate the expression of PGC1α, which is a transcription factor upstream of FNDC5. Both factors play an important role in ovarian function (16). Therefore, the aim of this study was to evaluate PGC1α-FNDC5 axis in granulosa cells derived from PCOS animal model, in hope of better understanding of the role of this gene in PCOS.

## Methods

### Generation of PCOS animal model:

25-day-old (12 gr) prepubertal female BALB/c mice were provided by the Animal Center of Tehran University of Medical Sciences. Mice were housed 3–4 *per* cage with standard laboratory conditions (12 *hr* in light and 12 *hr* in dark cycle, temperature-controlled facility at 25±1°*C*) ad libitum. All animal experimental procedures were approved by the Institutional Animal Care Committee of Tehran University of Medical Sciences. PCOS was induced by daily subcutaneous administration of 6 *mg* per 100 *g* bodyweight dehydroepiandrosterone (DHEA, Sigma-Aldrich) dissolved in 0.01 *ml* of 95% ethanol and mixed with 0.09 *ml* sesame oil for 20 consecutive days (experimental group: n=20). The control group consisting of mice received daily subcutaneous sesame oil injection for an equivalent length of time (vehicle group: n=20) and also those without any treatment (control group: n=20). Estrous cycles of all mice were determined by evaluating the cell types in vaginal smears (15). At the end of the experiment, animals were sacrificed by cervical dislocation. Ten ovaries of each group were removed and fixed overnight in 10% formalin for morphological studies. Blood sample was collected for assessment of serum steroid hormones (estradiol and progesterone).

### Morphological assessments:

To evaluate the histological changes in the ovary, the formalin-fixed ovaries of each group (DHEA and control groups) were embedded in paraffin. Embedded ovarian tissues were cut into 5 *μm* sections using microtome. Sections were deparaffinized with xylene and rehydrated in ethanol 60%. Subsequently, sections were washed in PBS and stained with hematoxylin and eosin (DAKO, CA, USA) and mounted on a glass slide for histomorphologic examinations of cyst formation in polycystic ovaries.

### Vaginal smears and estrous cycle determination:

The estrus cycle of mice was determined by microscopic cytological analysis of the predominant cell type in vaginal smears obtained daily using crystal violet staining (Sigma, CA, USA). The estrus cycle of mice is approximately 4–5 days, including proestrus, estrus, metestrus and diestrus (15). To obtain vaginal epithelial cells, sterile ddH2O was flashed at the opening of the vaginal canal with a sterile 200 *μL* tip several times and final flush was transferred on a glass slide and allowed to completely dry at room temperature before staining.

### Measurement of serum hormones:

After treatment for 20 days, blood samples were obtained by cardiac puncture from control and DHEA-treated mice and sera were separated for steroid hormones determination. The levels of 17-β-estradiol (E2) and progesterone (P) were determined by radioimmunoassay kit (Siemens, Dublin, Ireland) for each sample. Also, luteinizing hormone (LH) and Follicle-stimulating hormone (FSH) activities were performed with an ELISA method using Mouse LH and FSH ELISA Kits (Biocompare, USA). The hormonal determination was performed according to the manufacturer’s instructions.

### Isolation of granulosa cells:

Ovaries were removed from DHEA-treated mice and control mice and transferred to Hank’s balanced salt solution (HBSS; Life Technologies, MA, USA). Large preantral and antral follicles were punctured with a 25-gauge needle and isolated granulosa cells or a mixture of oocyte/granulosa cells were filtered by a 70 *μm* cell strainer (BD Falcon, MA, USA) to remove ovarian debris and small follicles. Then, granulosa cell/oocyte suspensions were filtered through a 40 *μm* cell strainer (BD Falcon, MA, USA) to separate granulosa cells from oocytes. The granulosa cells were collected by centrifugation at 300 *g* for 5 *min* at 4°*C*. Viability of isolated granulosa cells was determined by Trypan blue (Sigma, CA, USA) exclusion.

Identity and purity of isolated granulosa cells were judged using immunofluorescence staining for a specific marker of granulosa cells, follicle stimulating hormone receptor (FSHR). To stain with primary antibody to FSHR (Abcam, CA, UK), the cells were washed with PBS and fixed with 4% paraformaldehyde and non-specific binding sites were blocked. FITC-conjugated anti-rabbit IgG (Abcam, CA, UK), as a secondary antibody, was used after three times of washing with PBS for 1 *hr* at 4°*C*. Finally, the cells were rinsed in PBS and assessed under an Olympus BX 60 fluorescent microscopy (Olympus, Japan).

### RNA isolation and quantitative real-time PCR (qRT-PCR):

Total RNA was extracted from granulosa cells by the RNeasy Mini Kit (Qiagen, Germany) and treated with DNaseI to remove contaminating genomic DNA. cDNA synthesis was performed with 1 *μg* of total RNA utilizing oligo dt-primer with a cDNA Synthesis Kit (Thermo Scientific, USA). cDNA fragments were subjected to SYBR Green RT-PCR (TaKaRa, Japan) on an Applied Biosystems (Step one Plus; Applied Biosystems, USA) thermal cycler according to the manufacturer’s protocol. Relative expression of target genes was calculated using the ΔΔCT method and normalized to Gapdh gene expression level. Specific primer sequences were as previously described (17).

### Statistical analysis:

Microsoft Excel and SPSS (version 21) were used to analyze data. Data are expressed as mean±SEM (standard error of mean). Differences in all experiments among three groups were assessed using one-way ANOVA, Tukey’s Post Hoc test. The p<0.05 was considered statistically significant.

## Results

### Characterization of PCOS by histological analysis:

**Figure 1.**
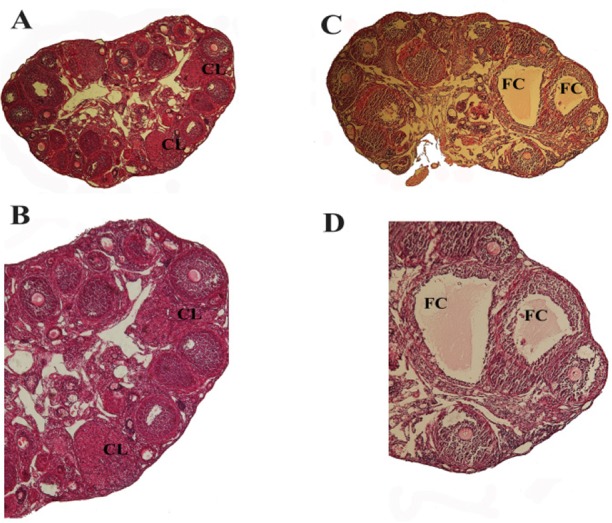
Morphological comparison of control and DHEA-treated PCOS mice ovaries. Control/vehicle ovary showed several follicles in different developmental stages. Corpus luteum (CL) structure is also shown (A, B); DHEA-treated PCOS mouse ovary showed various stage developing follicles but a high number of antral and preantral follicles and some large follicular cysts (FC) and/or hemorrhagic follicular cysts (HFC). The morphology of cysts is described by a thin layer of theca cells and a compacted formation of granulosa cells. Corpus luteum was absent in polycystic ovary (C, D). Scale bar: 50 *μm*

The ovarian sections of control and vehicle mice had general appearance of the tissue resembling the normal histology and the ovarian cortex enclosed large numbers of follicles in different stages of development with healthy oocytes (Figure 1A and 1B). Synchronized follicular development and abundant corpora lutea (CL) were observed which showed normal follicular maturation and ovulation in control mice. Also, the central medulla of control mice consisted of normal vessels network areas. Histological evaluation of the ovarian sections of DHEA-treated mice revealed distorted morphology. The peripheral cortex of ovaries showed an increase in the number of antral and atretic follicles which contained degenerated oocytes in DHEA-treated mice. DHEA treatment also yielded formation of follicular cysts which was characterized by a compacted formation of granulosa cells and thin layer of theca cells. Histological examination showed the absence of corpora lutea in PCOS ovary which may reveal disrupted follicular development and ovulation (Figure 1C and 1D). Increasing the size and number of arrested and cystic follicles, especially hemorrhagic follicular cysts (HFC) where small blood vessels were broken and allowed blood to fill the cyst, made the polycystic ovaries larger than control and vehicle groups (Figure 1C). Also, increased medullar area and enlarged vessels network were observed in PCOS ovaries.

### Determination of estrus cycle stage:

Since menstrual disturbance is a typical clinical feature of PCOS women, estrous cycles were examined in all groups of mice by evaluating the cell types in vaginal smears. Cytological examination revealed that both control and vehicle mice had normal estrous cyclicity and showed different stages of the cycle containing proestrus (round, nucleated epithelial cells), estrus (cornified squamous epithelial cells), metestrus (cornified squamous epithelial cells and predominance of leukocytes), and diestrus (predominance of leukocytes) (Table 1). In contrast, DHEA-treated mice were completely acyclic and remained in constant estrus cycle. Only control mice were used in the estrus cycle for further examination to eliminate the effect of estrous cycles on other measurements.

**Table 1.** Cytological features following vaginal smear represent each stage of estrous cycle

**Stage of estrous cycle**	**Nucleated epithelial cells**	**Squamous epithelial cells**	**Cornified squamous epithelial cells**	**Leukocytes**
**Proestrus**	+	-	-	-
**Estrus**	-	+	-	-
**Metestrus**	-	-	+	+
**Diestrus**	-	-	-	+

Proestrus, predominantly consisting of nucleated epithelial cells; estrus, with cornfield squamous epithelial cells; metestrus, consisting of cornified squamous epithelial cells and predominance of leukocytes; and diestrus, consisting predominantly of leucocytes

### Serum hormone levels:

**Figure 2.**
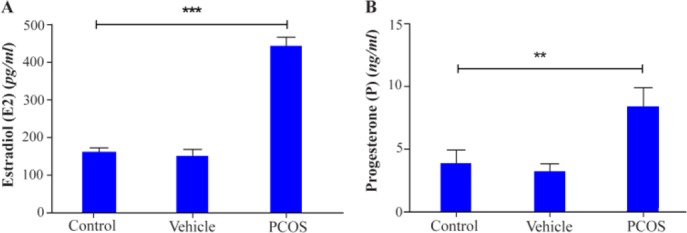
Serum (A) estradiol and (B) progesterone concentrations from control and DHEA-treated mice were evaluated by radioimmunoassay in mice (20 mice for each group). The serum levels of estradiol and progesterone were increased after treatment with DHEA. Asterisks indicate significant differences. **, p<0.01; ***, p<0.001, which was calculated with one-way ANOVA, Tukey’s Post Hoc test

To evaluate ovarian function after treatment with DHEA, serum estradiol and progesterone levels were evaluated by specific radioimmunoassay. Treatment with DHEA for 20 consecutive days increased both serum E2 and P levels in PCOS mice when compared with control and vehicle mice (p<0.001 and p<0.01, respectively) (Figure 2). On the other hand, LH, FSH and LH/FSH levels were assessed in PCOS mice vs control and vehicle groups. As observed LH, FSH and LH/FSH levels were increased in PCOS mice when compared with the counterparts of both control and vehicle groups (Table 2).

**Table 2.** Serum hormone levels in control, vehicle, and DHEA-treated PCOS mice

	**LH (IU/l)**	**FSH (IU/l)**	**LH/FSH**
**Control**	11±0.22	5.36±1.1	2.05±0.2
**Vehicle**	11.4±1.85	5.62±0.12	2.02±0.4
**DHEA**	23.5±0.63^*^	6.11±0.11^*^	3.85±0.22^*^

* Significant difference between OPCS mice (DHEA treated mice) with control and vehicle samples (p<0.05)

### Determining the identity of isolated cell:

**Figure 3.**
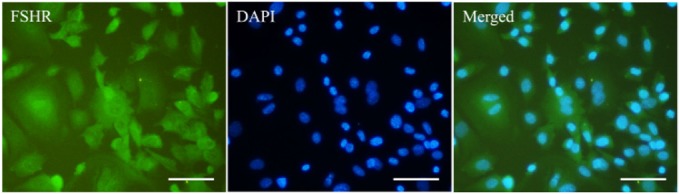
Immunostaining of isolated granulosa cells with FSHR antibody that represented FSHR (green) expression in these cells. Nuclei (blue) were counterstained using DAPI. Scale bar: 100 *μm*

Immunostaining of isolated cells showed the expression of FSHR, as a specific marker of granulosa cells (Figure 3).

### Expression levels of PGC1α and FNDC5:

**Figure 4.**
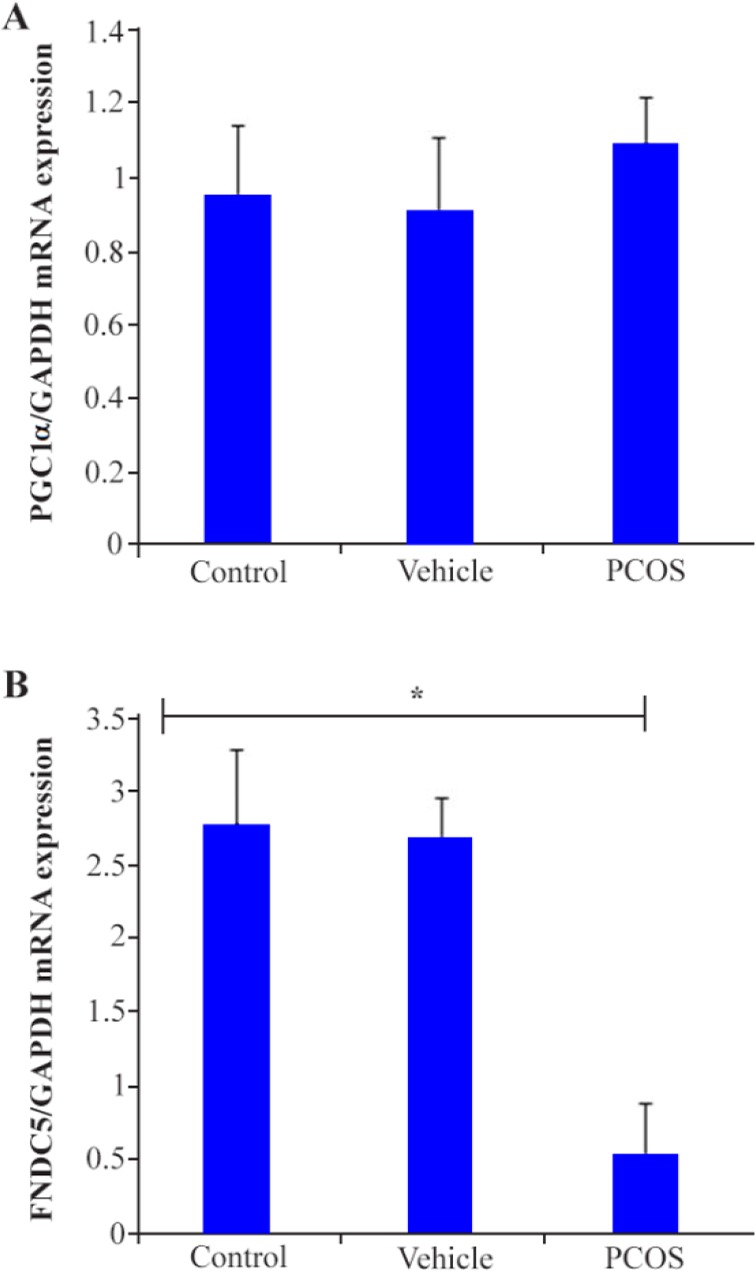
No significant difference in expression of PGC1α was observed between groups, (A) whereas, significant down regulation in FNDC5 transcripts was observed in granulosa cells of PCOS mice compared with the control (B). Star indicates significant difference between samples at p<0.05. The number of mice for each group was 20. One-way ANOVA, Tukey’s Post Hoc test was used for statistical analysis

Granulosa cells were isolated from antral and pre-antral cystic follicles of DHEA-induced PCOS mice and pre-antral and antral follicles of control and vehicle mice to evaluate expression level of PGC1α and FNDC5. There was no significant difference in expression of PGC1α whereas, a significant down regulation in FNDC5 transcripts was observed in granulosa cells of PCOS mice compared with the control (p<0.05) (Figure 4).

## Discussion

Considering the role of PGC1α/FNDC-5 in regulation of cellular metabolism and production of sex hormones (16) and the fact that levels of these hormones are altered in PCOS (18), the purpose of the study was to assess the expression of these genes in granulosa cells of PCOS mice animal model. Animal model was induced by treating mice with DHEA as already published (19). To verify that PCOS model was correctly induced, histological sections from ovaries were obtained and compared to the control group. In addition, vaginal smears were obtained to show that estrous cycle was interrupted in PCOS animal model. In addition, both secretory dynamics of estradiol (E2) and progesterone (P4) were also elevated. These together verify that PCOS animal model was produced. Our results revealed that the expression of FNDC5 in granulosa cells was significantly reduced compared to control. However, there was no significant difference in transcript level of PGC1α in PCOS mice and control group. Moreover, Jedrychowski et al. have shown that irisin was reduced significantly in follicular and serum of PCOS individuals (12). Recently, Zhoa et al. have shown a methylated type of PGC1α promoter found in the leukocytes of PCOS patients, but to our knowledge no previous study has assessed transcript level of PGC1α/FNDC5 axis in cumulus cells surrounding the oocytes. Our results are also in concordance with previous report describing serum irisin concentrations of women with PCOS which were lower compared to the controls (13). Unaltered expression of PGC1α, with concomitant reduced transcript level of FNDC5 in cumulus cells and reduced amount of irisin in follicular fluid in PCOS patient may suggest the expression of FNDC5 and its subsequent release and regulation of irisin by unidentified mechanism rather than through PGC1α. Previous studies have indicated that insulin infusion in PCOS women is responsible for an increase in serum irisin (20). Also, serum irisin concentrations of PCOS women have been reported to be higher compared to the controls and associated with serum levels of LH (21, 22). Inversely, a negative correlation has been reported to exist between serum LH level and circulatory irisin (12). Despite such discrepancies, there is no report on the origin of serum irisin in PCOS patients.

## Conclusion

Our data revealed that the axis of PGC1α/FNDC5 was disrupted in granulosa cells of PCOS animal model. As our report is the first study on assessment of granulosa cells derived FNDC5, to clarify the real molecular mechanism of such phenomenon, it should be investigated in further studies.
